# Risk-benefit analysis of isoniazid monotherapy to prevent tuberculosis in patients with rheumatic diseases exposed to prolonged, high-dose glucocorticoids

**DOI:** 10.1371/journal.pone.0244239

**Published:** 2020-12-31

**Authors:** Jun Won Park, Jeffrey R. Curtis, Hajeong Lee, Jung-Kyu Lee, Yeong Wook Song, Eun Bong Lee

**Affiliations:** 1 Division of Rheumatology, Department of Internal Medicine, Seoul National University College of Medicine, Seoul, Republic of Korea; 2 Division of Clinical Immunology & Rheumatology, University of Alabama at Birmingham, Birmingham, AL, United States of America; 3 Division of Nephrology, Department of Internal Medicine, Seoul National University College of Medicine, Seoul, Republic of Korea; 4 Division of Pulmonary and Critical Care Medicine, Department of Internal Medicine, Seoul Metropolitan Government Seoul National University Boramae Medical Center, Seoul, Republic of Korea; 5 Department of Molecular Medicine and Biopharmaceutical Sciences, Graduate School of Convergence Science and Technology, Seoul National University, Seoul, Republic of Korea; 6 Wide River Institute of Immunology, Seoul National University College of Medicine, Hongcheon, Republic of Korea; The University of Georgia, UNITED STATES

## Abstract

**Objective:**

To investigate the incidence of tuberculosis (TB) in patients with rheumatic diseases receiving high-dose glucocorticoids and to evaluate the preventive effect of isoniazid (INH).

**Methods:**

This study included 1618 treatment episodes of prolonged (≥4 weeks), high-dose steroids (≥30mg/day of prednisone) in 1160 patients. Of these, INH was administered in 152 (9.4%) treatment episodes (INH group), while others received no prophylaxis (control group). The high-risk subgroup (n = 92) was defined as patients with 1) incomplete adherence to treatment of previous TB, 2) positive interferon-γ release assay, and/or 3) linear/reticular fibrotic lesions on chest radiographs. Primary outcome was 1-year incidence of TB in each group.

**Results:**

During 1579.8 person-years, 21 cases of TB occurred. The high-risk subgroup showed a significantly higher TB incidence than the non-high-risk subgroup (Incidence rate ratio = 8.29). INH did not significantly affect the 1-year TB incidence in the whole population but numerically reduced it only in the high-risk subgroup [adjusted hazards ratio = 0.37 (95% CI, 0.002–5.10)]. The incidence of adverse drug reactions (ADRs) related to INH was 111.6 (89.3–137.9)/100 person-years, including one fatal occurrence of fulminant hepatitis. The number needed to treat (NNT) to prevent one case of TB was lower than the number needed to harm (NNH) for one case of severe ADR only in the high-risk subgroup (11 vs. 16).

**Conclusion:**

INH treatment to prevent TB might be effective in high-risk patients but has a risk of frequent ADRs, which limits its use in general practice in patients not at a high risk of developing TB.

## Introduction

Tuberculosis (TB) caused by *Mycobacterium tuberculosis* is an important healthcare problem globally. In 2016, 6.3 million new cases of TB and 1.3 million cases of TB-related death were reported among human immunodeficiency virus (HIV)-negative patients [1]. Although its incidence has been slowly decreasing since 2002, it is still a significant co-morbidity in patients with rheumatic diseases because the immunosuppressive agents used to treat rheumatic diseases increase the risk of TB [2]. Among the various immunosuppressive agents, high-dose glucocorticoids, a mainstay of the treatment of many rheumatic diseases, are particularly known to increase the TB risk [3]. However, because there have been no studies investigating the incidence of TB disease in such populations, it remains uncertain whether testing for latent tuberculosis infection (LTBI) and/or prophylactic treatment could be beneficial, especially in patients not being treated with tumor necrosis factor inhibitor or Janus kinase inhibitors, where routine TB screening is recommended [4, 5]. Furthermore, previous studies that investigated the efficacy of isoniazid (INH) prophylaxis in high-dose steroid users included a small number of patients and showed inconsistent results [6, 7]. Therefore, most national guidelines for TB prophylaxis, especially those relevant to rheumatic disease patients, do not thoroughly address this issue, which has led to highly variable practice among rheumatologists regarding the diagnosis and treatment of LTBI in patients with rheumatic diseases receiving high-dose steroids [8, 9].

To address this problem, we investigated the incidence of TB and its risk factors in a large, single-center cohort of patients with rheumatic diseases who underwent prolonged high-dose steroid treatment. In addition, we also analyzed the efficacy and safety of prophylactic INH monotherapy, so that we could conduct a precise risk-benefit assessment of this strategy in both high-risk and non-high-risk patients.

## Methods

### The incidence of TB and strategy for LTBI detection in South Korea

The incidence of TB in South Korea was 96 per 100,000 person-years in 2005 and decreased to 77 per 100,000 person-years in 2016. Bacillus Calmette-Guérin (BCG) vaccination at birth has been mandatory since 1965, and the rate of vaccination in children under 3 years was 65.7% in 1990 and 99.8% in 2013. In the 7^th^ Korean National Health and Nutrition Examination Survey in 2016, the prevalence of LTBI was 33.2% [95% confidence interval (CI), 30.9–35.6] [10, 11]. It was increasing with age, with 6.5% and 48.7% in the age group of 10–19 and 50–59, respectively.

Interferon-γ release assays (IGRAs) have been used to screen for LTBI at our institution since 2009. A recent revised national guideline for TB in South Korea recommends IGRA alone or an IGRA plus tuberculosis skin test (TST) for the diagnosis of LTBI in immunosuppressed patients. However, TST alone is not recommended due to its low specificity in BCG-vaccinated patients. Of note, this guideline does not address the risk of developing TB or the need for LTBI evaluation in patients with rheumatic diseases receiving high-dose steroids due to lack of the robust evidence [12].

### Patients and clinical data

First, we captured clinical situations in which patients with a rheumatic disease were treated with high-dose glucocorticoids for more than 4 consecutive weeks (defined as a treatment episode) between January 2004 and December 2017 from the electronic medical database at Seoul National University Hospital. High-dose glucocorticoid treatment was defined as ≥30 mg/day of prednisone or equivalent [13]. The medical classifications used for case identification of rheumatic diseases according to the 10^th^ revision of the International Statistical Classification of Diseases and Related Health Problems (ICD-10) are listed in the online supplementary text. Patients younger than 18 or with a history of HIV infection, current cancer, solid organ transplantation, or active TB disease requiring treatment at the start of high-dose glucocorticoids were excluded. Patients who concomitantly received tumor necrosis factor inhibitor during the observation period were also excluded. All captured treatment episodes were then divided into two groups (INH and control groups) according to whether a patient received INH with the high-dose steroid treatment. We applied ‘intention-to-treat’ design in which any administration of INH during the observation period was necessary to determine whether an episode was to be included in the INH or unexposed (control) group.

The baseline date was defined as the first day of starting INH (in the INH group) or high-dose steroids (in the control group). Each patient remained on high-dose steroids for at least 4 weeks after the baseline date. The observation period for each treatment episode was 1 year from the baseline date unless TB disease or censoring events (death or loss to follow-up, defined as no follow-up visits for longer than 12 weeks from the previous hospital visit) occurred. Therefore, the study could include multiple treatment episodes from single patients if they re-started prolonged high-dose steroid treatment more than 1 year after the relevant baseline date. The definition and observation of treatment episodes are described in S1 Fig.

Demographic and clinical features at baseline, such as the initial steroid dose, concomitant immunosuppressant treatment, lymphocyte count, and chest radiographs, were collected. Data regarding the cumulative glucocorticoid dose during the 6 months prior to the baseline date were also collected. High-risk treatment episodes (High-risk subgroup) were defined as those with at least one of the following clinical factors at baseline; 1) incomplete adherence to treatment of previous TB infection, 2) a positive IGRA result, and 3) linear or reticular fibrotic lesions on chest radiographs, a pattern that is consistent with old TB lesion [14, 15].

The primary outcome was the 1-year incidence of TB in each group. The secondary outcome was the incidence of adverse drug reactions (ADRs) related to INH therapy. All potential ADRs in the INH group were reviewed and assigned a causality based on the chronology, known patterns of adverse effects associated with INH, and known effects of INH withdrawal [16]. Events with a probable/likely or certain causality were ultimately defined as ADRs in this study. The severity of ADRs was assessed according to the Common Terminology Criteria for Adverse Events (CTCAE), version 5.0 and severe ADR was defined as that with grade 3 or higher.

This study was carried out in accordance with the Declaration of Helsinki and was approved by the Institutional Review Board of Seoul National University Hospital (No 1508-050-694). Patient consent was waived due to the retrospective nature of the study and the data were analyzed anonymously.

### Detection of TB

TB was diagnosed when a patient had both clinical and microbiological evidence of TB. To detect all cases of TB, data relating to acid-fast bacilli staining/culture and TB polymerase chain reaction from the included patients were reviewed. If a patient showed a positive result in at least one of the above tests, his or her medical records were reviewed to ascertain whether he or she had clinical features suggesting TB disease, and if so, anti-tuberculosis treatment was administered. A positive TB polymerase chain reaction result in the absence of clinical evidence of TB was not considered a true case of TB.

### Isoniazid treatment to prevent TB

Because of the lack of universal recommendations regarding the evaluation of LTBI in patients with rheumatic diseases receiving high-dose steroids, TST and/or IGRA were not routinely performed during the observation period. In addition, according to the national guideline on TB in South Korea, treatment for LTBI in chronic steroid users could be individually considered according to patient’s clinical factors [12]. Therefore, the selection of patients who would be treated with INH and its duration were at the discretion of the treating physician and mainly based on the underlying disease, the initial steroid dose, and concomitant immunosuppressant treatment. Patients who received prophylactic treatment received 6 mg/kg INH (up to a maximum dose of 300 mg) once a day with pyridoxine replacement. There were no treatment episodes in which rifampicin or the combination of INH plus rifampicin was used for prophylaxis because rifampicin shows a significant drug-drug interaction with glucocorticoids, and decreases its therapeutic effect [17]. Once-weekly INH and rifapentine for 12 weeks regimen was not approved for LTBI treatment in chronic steroid users in South Korea, and therefore, was not used in the study population.

### Statistical analysis

Continuous or dichotomous baseline data were compared using Student’s t-tests or Chi-square tests, as appropriate. The incidence of TB between the two groups was compared using Poisson regression. The influence of clinical factors, including prophylactic INH, on outcome was analyzed using the Cox proportional hazards model. The hazards ratio (HR) was adjusted for clinical factors that showed a relevant association (P < 0.1) with outcome in univariable analyses. The multivariable model was further adjusted for intra-cluster correlations using grouped sandwich variance estimates because some patients contributed multiple treatment episodes. If an outcome variable showed a complete separation between the two groups, Firth’s penalized maximum likelihood was used for analysis [18]. All statistical analyses were performed using R V.3.3.1 software, and a P-value <0.05 was considered statistically significant.

## Results

### Baseline characteristics

A total of 1618 treatment episodes from 1160 patients were analyzed in this study. Overall, mean (standard deviation, SD) age of the patients was 42.3 (15.6) years and 492 (30.4%) patient were male. Systemic lupus erythematosus (SLE) was the most common underlying rheumatic disease (800/1618, 49.4%). Patients with SLE were more likely to be younger (49.0 vs. 35.6 years), be female (57.6% vs. 71.9%), and more frequently received steroid pulse therapy and other immunosuppressants such as cyclophosphamide and mycophenolate mofetil (S1 Table).

Prophylactic INH was administered with high-dose glucocorticoids in 152 (9.4%) treatment episodes, for a mean (SD) duration of 185 (153) days. In the INH group, INH was started along with high-dose steroids in most cases (149/152, 98.0%). In the remaining three treatment episodes, INH was delayed for 3–4 weeks. However, no incidences of TB occurred during the delay. The schema for inclusion in this study is shown in S2 Fig.

The baseline characteristics of the patients are presented in Table 1. Briefly, patients in the INH group were older (45.1 vs. 42.1 years) and were more likely to have SLE or microscopic polyangiitis as their underlying disease. By contrast, the proportion of patients with rheumatoid arthritis (1.3% vs. 4.8%) or Behcet disease (2.0% vs. 13.3%) was significantly lower in the INH group. In addition, more patients in the INH group concomitantly received oral cyclophosphamide, cyclophosphamide pulse therapy (intravenous), or steroid pulse therapy compared with the control group. The mean daily steroid dose (based on prednisone) that patients received during the 6 months prior to the baseline date was also higher in the INH group (9.9 vs. 12.6 mg/day), which suggests that the patients in the INH group were more immunosuppressed.

**Table 1:** Baseline^a^ characteristics of the treatment episodes (n = 1618).

(n = number of treatment episodes)	Control group	INH group	*P*-value
	(n = 1466)	(n = 152)	
Age, year, mean (SD)	42.1 (15.5)	45.1 (17.1)	0.040
Male sex, n (%)	442 (30.2)	50 (32.9)	0.484
Disease duration, year, mean (SD)	3.2 (4.1)	2.0 (3.2)	<0.001
Underlying diseases, n (%)			
Systemic lupus erythematosus, n (%)	714 (48.7)	86 (56.6)	0.065
Systemic sclerosis, n (%)	33 (2.3)	2 (1.3)	0.451
Dermatomyositis, n (%)	131 (8.9)	20 (13.2)	0.089
Polymyositis, n (%)	71 (4.8)	3 (2.0)	0.107
GPA, n (%)	34 (3.7)	5 (3.3)	0.805
MPA, n (%)	13 (0.9)	8 (5.3)	<0.001
EGPA, n (%)	45 (3.1)	7 (4.6)	0.307
Polyarteritis nodosa, n (%)	26 (1.8)	4 (2.6)	0.455
Rheumatoid arthritis, n (%)	70 (4.8)	2 (1.3)	0.049
Adult-onset Still’s disease, n (%)	39 (2.7)	3 (2.0)	0.612
Behcet’s disease, n (%)	195 (33.3)	3 (2.0)	<0.001
Primary Sjogren’s syndrome, n (%)	8 (0.5)	1 (0.7)	0.859
Others, n (%) ^b^	54 (3.7)	7 (4.6)	0.570
High-risk for LTBI, n (%) ^c^	76 (5.2)	16 (10.5)	0.007
Incomplete adherence to treatment of previous TB infection	19/1466 (1.3)	3/152 (2.0)	0.492
Positive IGRA result	12/161 (7.5)	4/26 (15.4)	0.063
Linear or reticular fibrotic lesions on chest radiographs	56/1350 (4.1)	9/148 (6.1)	0.273
Steroid pulse treatment, n (%)	226 (15.4)	59 (38.8)	<0.001
Oral cyclophosphamide, n (%)	65 (4.4)	19 (12.5)	<0.001
Cyclophosphamide pulse treatment, n (%)	130 (8.9)	34 (22.4)	<0.001
Mycophenolate mofetil, n (%)	112 (7.6)	5 (3.3)	0.049
Cyclosporine, n (%)	94 (6.4)	15 (9.9)	0.106
Methotrexate, n (%)	98 (6.7)	8 (5.3)	0.500
Mean steroid dose used during the prior 6 months, mg/day, mean (SD) ^d^	9.9 (10.0)	12.6 (9.5)	0.002
Baseline lymphopenia, n (%) ^e^	335 (22.9)	57 (37.5)	<0.001

IGRA, interferon-γ release assay; INH, isoniazid; LTBI, latent tuberculosis infection; GPA, granulomatosis with polyangiitis; MPA, microscopic polyangiitis; EGPA, eosinophilic granulomatosis with polyangiitis; PD, prednisone; SD, standard deviation.

^a^ The baseline date was defined as the day on which INH prophylaxis (INH group) or high-dose steroids (control group) were started.

^b^ Including polymyalgia rheumatica, Takayasu’s arteritis, temporal arteritis, and relapsing polychondritis.

^c^ Including an incomplete adherence to treatment of previous TB infection, a positive IGRA result, and/or the presence of linear or reticular fibrotic lesions on chest radiographs.

^d^ Based on the dose of prednisone.

^e^ Defined as <800 lymphocytes per microliter.

Among the entire population, the number of patients with a history of incomplete treatment for previous TB infection was 22 (1.4%). IGRA was performed at baseline in 187 (11.6%) treatment episodes, with a positive rate of 8.6% (16/187). Among the 1498 (92.6%) treatment episodes with available chest radiographs at baseline, 65 (4.3%) showed linear or reticular fibrotic lesions on chest radiographs. Finally, the number of patients in the high-risk subgroup was 92 (5.7%), and 16 (17.4%) of them received prophylactic INH.

### Incidence of TB disease and its risk factors

**Fig 1:**
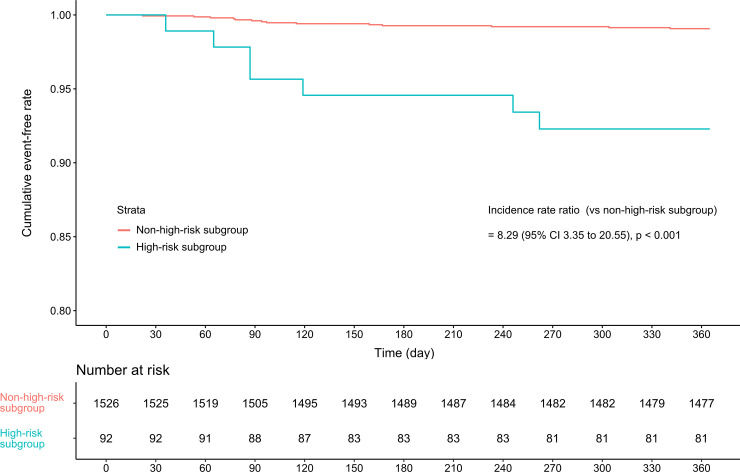
Kaplan–Meier curves indicating the 1-year tuberculosis incidence according to the presence of high-risk factors.

During the observation period, 96.3% (1557/1618) episodes completed the 1-year follow-up and the mean (SD) follow-up duration was 356.5 (46.7) and 355.6 (48.5) days in the control and the INH groups, respectively. There were 21 cases of TB occurred, for an incidence of 1329 (95% CI, 823–2032) per 100,000 person-years. The clinical features of the 21 cases at baseline and at the time of TB occurrence are summarized in S2 and S3 Tables, respectively. SLE was the most frequent underlying rheumatic disease in the patients who developed TB disease (15/21, 71.4%), and the mean duration between the baseline date and the diagnosis of TB was 131.9 (92.2) days. Fifteen patients (71.4%) had pulmonary TB. Extrapulmonary involvement and miliary TB occurred in seven (33.3%) and four (19.0%) patients, respectively. There were four cases of multidrug-resistant TB. In the other cases, 14 (66.7%) patients were initially treated with the standard combination regimen of INH, rifampicin, ethambutol, and pyrazinamide (S3 Table). The incidence (per 100,000 person-years) of TB in the high-risk subgroup was significantly higher than that in the non-high-risk subgroup [8245 (seven cases/84.9 person-years) vs. 936 (14 cases/1495.0 person-years)] (Fig 1).

Univariable Cox analysis also showed that presence in the high-risk subgroup was the most important risk factor associated with TB disease [crude HR = 8.62 (95% CI, 3.48–21.35)]. In addition, underlying SLE, a higher mean steroid dose used during the 6 months prior to the baseline date, duration of high-dose steroid treatment from the baseline, and concomitant steroid pulse treatment were also associated with increased risk of TB. However, the initial steroid dose and concomitant treatment of immunosuppressants such as cyclophosphamide, mycophenolate mofetil, cyclosporine and methotrexate did not show a relevant association with the outcome. In the multivariable analysis, SLE, longer duration of high-dose steroid treatment, and high-risk subgroup were significantly associated with increased risk of TB (Table 2).

**Table 2:** Clinical factors associated with the 1-year TB incidence during the observation period.

	Univariable analysis		Multivariable analysis ^a^	
	HR (95% CI)	*P-*value	Adjusted HR (95% CI)	*P-*value
Age (per 5-year increment)	0.95 (0.82–1.10)	0.475	^b^	
Male sex	1.40 (0.51–3.82)	0.513	^b^	
Disease duration (per 5-year increment)	0.95 (0.55–1.64)	0.845	^b^	
SLE	2.54 (0.99–6.55)	0.054	3.10 (1.24–7.75)	0.016
High-risk subgroup	8.62 (3.48–21.35)	<0.001	13.95 (5.61–34.64)	<0.001
Initial steroid dose at baseline (≥60 mg/day of prednisone vs. a lower dose) ^c^	0.92 (0.39–2.18)	0.924	^b^	
Concomitant oral cyclophosphamide	0.42 (0.003–3.04)	0.485	^b^	
Concomitant cyclophosphamide pulse	1.47 (0.43–5.00)	0.535	^b^	
Concomitant mycophenolate mofetil	2.16 (0.63–7.38)	0.219	^b^	
Concomitant cyclosporine	0.69 (0.09–5.17)	0.718		
Concomitant methotrexate	0.72 (0.10–5.33)	0.747		
Concomitant steroid pulse	2.38 (0.96–5.89)	0.061	1.55 (0.63–3.80)	0.336
Mean steroid dose used during the prior 6 months, mg/day ^d^	1.04 (1.01–1.06))	0.007	1.02 (0.99–1.05)	0.201
Duration of high-dose steroid treatment, day	1.01 (1.006 to 1.013)	<0.001	1.01 (1.004 to 1.013)	<0.001
Baseline lymphopenia ^e^	1.26 (0.49–3.25)	0.632	^b^	

CI, confidence interval; HR, hazard ratio; SLE, systemic lupus erythematosus; TB, tuberculosis.

^a^ Model included the clinical factors that showed a significant association (*P* < 0.1) in univariable analyses, and was adjusted for clustering.

^b^ Not included in the multivariable model as a covariate.

^c^ Dose was calculated after excluding the dose of the concomitant steroid pulse treatment.

^d^ Based on the dose of prednisone.

^e^ Defined as <800 lymphocytes per microliter.

### Prophylactic effectiveness of INH treatment

**Fig 2:**
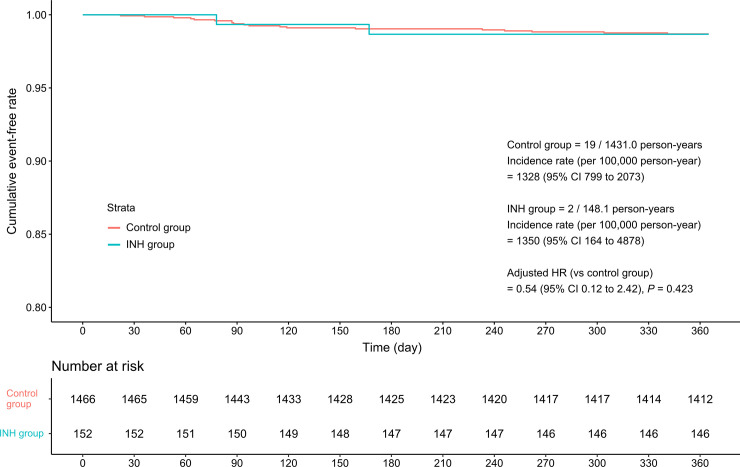
Kaplan–Meier curves indicating the 1-year tuberculosis incidence in the control and INH groups.

Two patients in the INH group developed TB, 72 and 169 days after the baseline date, respectively. One of them developed pulmonary TB by multidrug-resistant M. tuberculosis and both of them had been received INH with a good compliance by the time of TB diagnosis. The incidence rate (per 100,000 person-year) of TB was 1328 (19 cases/1431.0 person-years) in the control group and 1350 (two cases/148.1 person-years) in the INH group, which was not a significant difference (Fig 2). This result was consistent after adjusting for clinical factors associated with the 1-year incidence of TB disease [adjusted HR = 0.54 (0.12–2.42)].

The prophylactic effect of INH was further investigated after stratification of the treatment episode by the presence of high-risk factors at baseline. In the subgroup of patients without risk factors, INH treatment did not significantly influence the 1-year TB incidence [adjusted HR = 1.01 (0.20–5.08)]. In the high-risk subgroup, INH tended to reduce the risk of TB disease, but this difference was not statistically significant [adjusted HR = 0.37 (0.002–5.10)] (Table 3). A similar result was obtained when the Cox regression analysis was performed after excluding the three treatment episodes, in which the administration of prophylactic INH was delayed 3–4 weeks after the initiation of high-dose glucocorticoids (adjusted HR = 0.55 (0.12–2.50).

**Table 3:** The effect of prophylactic INH on the 1-year incidence of TB according to the presence of risk factors.

	Non-high-risk subgroup (n = 1526)	High-risk subgroup (n = 92) ^a^
Control group, n	1390	76
INH group, n	136	16
Number of TB cases / Observation period in the control group	12 / 1362.2 person-years	7 / 69.5 person-years
Number of TB cases / Observation period in the INH group	2 / 132.7 person-years	0 / 13.5 person-years
Crude hazards ratio of the INH group (95% CI)	0.76 (0.38–7.62)	0.31 (0.002–2.46)
Adjusted hazards ratio of the INH group (95% CI)	1.01 (0.20–5.08) ^b^	0.37 (0.002–5.10) ^c^

CI, confidence interval; INH, isoniazid; TB, tuberculosis.

^a^ Firth penalized maximum likelihood was used due to complete separation of outcome.

^b^ Included concomitant steroid pulse, duration of high-dose steroid treatment from the baseline and cumulative steroid dose as covariates, and was also adjusted for clustering.

^c^ Included age, male sex, duration of high-dose steroid treatment from the baseline and mean steroid dose used during the prior 6 months as covariates, and was also adjusted for clustering.

### Safety of INH treatment

During a total of 77.0 person-years of observation in the INH group, 86 ADRs occurred in the 68 treatment episodes, with an incidence of 111.6 (89.3–137.9) per 100 person-years (Table 4). Most ADRs were of mild-to-moderate severity, and 25 patients (36.8%) discontinued INH treatment. Increased serum alanine/aspartate aminotransferase (AST/ALT) level was the most common ADR [50.6 (36.0–69.2)/100 person-years)], followed by peripheral neuropathy [33.8 (22.1–49.5)/100 person-years] and gastrointestinal discomfort [13.0 (6.2–23.9)/100 person-years]. AST/ALT elevation more frequently occurred with increasing age (OR = 1.03, 95% CI 1.001–1.05) and with increasing cumulative dose of previously used steroids [OR = 1.0002, (95% CI 1.00002–1.0005)]. Peripheral neuropathy occurred more often in patients with concomitant cyclosporine treatment [OR 5.04 (1.44–17.60)]. In contrast, patients with higher dose of initial steroid (≥ 60mg/day of prednisolone or equivalent) were less likely to have the ADR [OR 0.41 (0.17 to 1.04)] (S4 Table).

**Table 4:** The incidence of adverse drug reactions caused by prophylactic INH.

	Number of cases ^a^	Incidence rate (95% CI) ^b^
Mild-to-moderate adverse drug reactions	85	110.4 (88.2 to 136.5)
AST/ALT elevation	31	40.3 (27.4 to 57.2)
Peripheral neuropathy	26	33.8 (22.1 to 49.5)
GI discomfort	10	13.0 (6.2 to 23.9)
Skin rash	6	7.8 (2.9 to 17.0)
Thrombocytopenia	5	6.5 (2.1 to 15.2)
Anemia	1	1.3 (0.03 to 7.2)
Leukopenia	1	1.3 (0.03 to 7.2)
Anorexia	2	2.6 (0.3 to 9.4)
Others ^c^	3	3.9 (0.8 to 11.4)
Severe adverse drug reactions ^d^	1	1.3 (0.03 to 7.2)
Fulminant hepatitis	1	1.3 (0.03 to 7.2)

ALT, alanine aminotransferase; AST, aspartate aminotransferase; CI, confidence interval; GI, gastrointestinal; INH, isoniazid; NA, not applicable.

^a^ The total observation period was 77.0 person-years for 152 episodes.

^b^ Rate per 100 person-years.

^c^ Including eosinophilia (n = 1), pruritus (n = 1), and general weakness (n = 1).

^d^ Occurred in the high-risk subgroup.

There was one case of fulminant hepatitis in a patient in the high-risk subgroup, and the patient ultimately expired due to progression to liver failure.

### Risk-benefit assessment of INH treatment

In the entire patient population, the number needed to treat (NNT) to prevent one case of TB was a negative value because the crude incidence rate was slightly higher in the INH group. The number needed to harm (NNH) to cause one ADR of any severity was 2 (1.6–2.1), and the NNH to cause one severe ADR was 152 (51.5–ꝏ). In the high-risk subgroup, the corresponding NNT was 11 (6.4–36.9), whereas the NNHs to cause any ADR or any severe ADR were 2 (1.3–3.9) and 16 (5.5–ꝏ), respectively. By contrast, in the non-high-risk subgroup, the NNT was negative, while the NNH for any ADR was 3 (1.9–2.8). NNH for any severe ADR in non-high-risk subgroup could not be calculated because there was no case of severe ADR in this subgroup.

### Sensitivity analysis

Since some baseline characteristics between the control and the INH groups were significantly different, we applied inverse probability of treatment weights (IPTW) to minimize this imbalance and repeated the main analysis (S3 Fig). The effect of INH on 1-year TB risk was not changed in this analysis [adjusted HR 0.85, (0.20–3.60)]. In addition, since the reactivation of TB occurs predominantly in the first two years, we also performed the same analysis using 2-year observation period [19]. In this analysis, a total of 1538 treatment episodes were included and 23 TB disease occurred, with an incidence rate of 809 (513–1244) per 100,000 person-years. Most of TB disease occurred within first 1-year period, suggesting that the effect of prolonged, high-dose steroid treatment on the risk of TB disease is decreasing after one year. INH did not decreased the incidence of TB disease, which was consistent with that in the original analysis [adjusted HR 0.49 (0.1–2.25)] (S3 Fig). Finally, because some patients in the non-high-risk subgroup was not fully evaluated on whether they had had all high-risk factor, this subgroup were actually composed of two different population; episodes with low-risk and with unknown-risk. Therefore, we estimated the effect of heterogeneity after excluding high-risk subgroup. Although there was no TB disease occurred in the low-risk subgroup, the effect of group-difference on 1-year incidence of TB disease was not significantly different [HR 2.82, (95% profile likelihood ratio 0.37–362.3, P = 0.397)]. Interaction between the group-difference and the effect of INH was also statistically insignificant (P = 0.525). This suggests that the result of the main analysis was less likely to be severely biased by the heterogeneity between the low- and unknown-risk subgroups. In the subgroup of unknown-risk treatment episodes, INH treatment did not show any significant effect on the risk for TB disease [adjusted HR 0.89 (0.16–4.93)] (S5 Table).

## Discussion

Systemic glucocorticoid therapy is an important treatment option for many rheumatic diseases, but it is also a major cause of opportunistic infections, including TB [3, 20, 21]. However, there have been few epidemiologic reports investigating the incidence of LTBI or providing a risk-benefit assessment for TB prophylaxis in patients with rheumatic diseases receiving high-dose steroids.

In the present study, the incidence of TB in patients with rheumatic diseases receiving prolonged, high-dose steroids was 1329 (95% CI, 823–2032) per 100,000 person-years, which was significantly higher than that of the general population in South Korea (77 per 100,000 person-years in 2016) [10]. This result is consistent with previous reports suggesting that impaired cellular immunity caused by steroid treatment significantly increases the risk of TB [7, 22]. However, INH treatment to prevent TB did not reduce its 1-year incidence, contrary to its proven efficacy in HIV-positive or transplant patients [23–26].

In this study, SLE showed an increased risk for TB, which is in line with many previous reports [27–29]. It was partially attributable to the fact that patient with SLE were more likely to receive steroid pulse therapy and additional immunosuppressive agents. However, the effect remained significant after adjustment for these factors, suggesting that SLE and TB share a genetic background that predispose to disease. Interestingly, a recent meta-analysis showed that human leukocyte antigen (HLA)-DRB1*15, which is significantly associated with SLE in different ethnic groups, was an important genetic risk factor for TB [30, 31]. In addition, previous studies also suggested that *Mycobacterium tuberculosis* can lead to autoimmunity by molecular mimicry between microbial antigen and host DNA [32, 33].

Presence in the high-risk subgroup, defined by incomplete adherence to treatment of previous TB infection, a positive IGRA result, and/or radiographic evidence of previous pulmonary TB, was the most important risk factor for TB, which suggests that reactivation of latent TB is a major cause of overt TB. Although current guidelines suggest that evaluation for LTBI by IGRA and/or TST before high-dose glucocorticoid treatment be considered, this is not based on robust evidence [9, 12]. There was, therefore, limited evaluation for LTBI in our study population. Furthermore, the diagnostic utility of TST in such patients is limited, because its sensitivity and specificity are significantly influenced by previous steroid use and BCG vaccination [34–36]. Although some studies reported the superior specificity of IGRA compared with TST, its diagnostic performance can also be impaired in patients receiving steroid treatment [37–40]. Therefore, defining the patient group in which prophylactic INH treatment could be beneficial should be based on a comprehensive evaluation consisting of careful evaluation of patient history, chest radiographs, and laboratory tests. In fact, the 1-year incidence of TB was similar in the control and INH groups, suggesting that non-selective application of INH treatment in the study population may not effective in preventing TB. By contrast, a subgroup analysis performed in the high-risk subgroup showed that INH treatment numerically reduced the 1-year incidence of TB. However, since the number of patients in this subgroup was small, the efficacy of INH prophylaxis in this population should be re-evaluated in future larger studies.

To determine whether prophylactic INH treatment is beneficial to patients, its safety profile is also an important consideration. In this context, it is of note that INH treatment was associated with frequent ADRs (including one fatal case of hepatitis), which resulted in a high discontinuation rate. A risk-benefit analysis also showed that the NNT for INH treatment was negative value in the whole population, suggesting low benefit. These results suggest that risk of ADRs for non-selective application of INH prophylaxis in patients with rheumatic diseases receiving prolonged high-dose steroids outweighs its potential benefit. By contrast, the NNT in the high-risk subgroup was slightly lower than the risk of severe ADRs, emphasizing again that INH prophylaxis should be considered only in this population.

This study has some limitations. First, because this study is retrospective design, some baseline characteristics between the control and the INH groups were significantly different, which leads to selection bias. We showed that the effect of INH was not changed after applying IPTW. However, the presence of unmeasured confounders such as patient’s compliance to INH cannot be solved without randomization. Second, the number of patients in the high-risk subgroup was small, and the screening criteria used to define this subgroup (e.g., IGRA testing) was not uniformly performed in the whole population, which reduces the statistical power of the study. Therefore, the prophylactic effectiveness of INH treatment should be further evaluated in future large-scale studies in which all included patients undergo IGRA test. Furthermore, since the number of TB cases was also small, it is possible that some clinical factors associated with increased risk for TB could not be significant in this study due to inadequate statistical power. Third, South Korea is a country of intermediate TB burden, so our results may not be applicable to other countries with different TB burdens, although our finding of minimal or no benefit in non-high-risk patients is presumably generalizable. Finally, mean duration of INH administration in the INH group was approximately 6 months, which was shorter than recommended for the treatment of LTBI. Therefore, prophylactic effect of INH could be underestimated in this study [41].

## Conclusion

In conclusion, this study showed the incidence of TB and the prophylactic effect of INH treatment in patients with rheumatic diseases receiving prolonged, high-dose glucocorticoid treatment. INH treatment to prevent TB might be effective in high-risk patients. However, the high incidence of ADRs limits its utility in non-high-risk patients.

## Supporting information

S1 Fig The definition and observational scheme of the treatment episode. (DOCX)

S2 Fig Flow of inclusion in this study. (DOCX)

S3 Fig Standardized mean differences (SMD) of baseline features before and after applying IPTW. (DOCX)

S4 Fig Effect of INH treatment on 2-year incidence of TB disease. (DOCX)

S1 Table Baseline characteristics of the patients with systemic lupus erythematosus (SLE) and other rheumatic diseases. (DOCX)

S2 Table Summary of the 21 TB cases occurred during the observation period. (DOCX)

S3 Table Clinical features of the 21 TB disease cases at diagnosis. (DOCX)

S4 Table Clinical factors associated with AST/ALT elevation and peripheral neuropathy during INH treatment. (DOCX)

S5 Table Incidence of TB disease and effect of INH treatment in the low-risk- and unknown-risk-subgroups. (DOCX)

S1 Text ICD-10 codes for detection of treatment episodes in patients with rheumatic diseases. (DOCX)
