# Supplementary material for: Risk-benefit analysis of isoniazid monotherapy to prevent tuberculosis in patients with rheumatic diseases exposed to prolonged, high-dose glucocorticoids
Source: PLoS One. 2020 Dec 31;15(12):e0244239. doi: 10.1371/journal.pone.0244239 (PMC7774985; doi:10.1371/journal.pone.0244239)
Supplement: S1 Fig — (DOCX) [file pone.0244239.s001.docx]

**S1 Fig.** The definition and observational scheme of the treatment episode
