# Supplementary material for: Risk-benefit analysis of isoniazid monotherapy to prevent tuberculosis in patients with rheumatic diseases exposed to prolonged, high-dose glucocorticoids
Source: PLoS One. 2020 Dec 31;15(12):e0244239. doi: 10.1371/journal.pone.0244239 (PMC7774985; doi:10.1371/journal.pone.0244239)
Supplement: S1 Table — (DOCX) [file pone.0244239.s005.docx]

**S1 Table.** Baseline characteristics of the patients with systemic lupus erythematosus (SLE) and other rheumatic diseases

| (n = number of treatment episodes) | No SLE  (n = 818) | SLE  (n = 800) | *P*-value |
| --- | --- | --- | --- |
| Age, year, mean (SD) | 49.0 (15.4) | 35.6 (12.6) | <0.001 |
| Male sex, n (%) | 347 (42.4) | 145 (18.1) | <0.001 |
| Disease duration, year, mean (SD) | 2.1 (3.6) | 4.0 (4.3) | <0.001 |
| High-risk for LTBI, n (%) ^a^ | 65 (7.9) | 27 (3.4) | <0.001 |
| Steroid pulse treatment, n (%) | 99 (12.1) | 186 (23.2) | <0.001 |
| Oral cyclophosphamide, n (%) | 81 (8.1) | 18 (2.2) | <0.001 |
| Cyclophosphamide pulse treatment, n (%) | 40 (4.9) | 124 (15.5) | <0.001 |
| Mycophenolate mofetil, n (%) | 15 (1.8) | 102 (12.8) | <0.001 |
| Cyclosporine, n (%) | 82 (10.0) | 27 (3.4) | <0.001 |
| Methotrexate, n (%) | 81 (9.9) | 25 (3.1) | <0.001 |
| Mean steroid dose used during the prior 6 months, mg/day, mean (SD) ^b^ | 397 (48.5) | 483 (60.4) | <0.001 |
| Baseline lymphopenia, n (%) ^c^ | 118 (14.4) | 274 (34.2) | <0.001 |

IGRA, interferon-γ release assay; INH, isoniazid; LTBI, latent tuberculosis infection; GPA, granulomatosis with polyangiitis; MPA, microscopic polyangiitis; EGPA, eosinophilic granulomatosis with polyangiitis; PD, prednisone; SD, standard deviation.

^a^ Including an incomplete adherence to treatment of previous TB infection, a positive IGRA result, and/or the presence of linear or reticular fibrotic lesions on chest radiographs.

^b^ Based on the dose of prednisone.

^c^ Defined as <800 lymphocytes per microliter.
