# Supplementary material for: Risk-benefit analysis of isoniazid monotherapy to prevent tuberculosis in patients with rheumatic diseases exposed to prolonged, high-dose glucocorticoids
Source: PLoS One. 2020 Dec 31;15(12):e0244239. doi: 10.1371/journal.pone.0244239 (PMC7774985; doi:10.1371/journal.pone.0244239)
Supplement: S2 Table — (DOCX) [file pone.0244239.s006.docx]

**S2 Table.** Summary of the 21 TB cases occurred during the observation period

| Age, year, mean (SD) | 39.8 (14.5) |
| --- | --- |
| Male sex, n (%) | 5 (23.8) |
| Disease duration, year, mean (SD) | 2.8 (3.8) |
| Underlying rheumatic disease, n (%) |  |
| Systemic lupus erythematosus | 15 (71.4) |
| Dermatomyositis | 1 (4.8) |
| EGPA | 1 (4.8) |
| Polyarteritis nodosa | 1 (4.8) |
| Behcet’s disease | 1 (4.8) |
| Others^*^ | 2 (9.6) |
| High-risk subgroup, n (%) | 7 (33.3) |
| IGRA positive | 0 (0.0) |
| Linear or reticular fibrotic lesions on chest radiographs. | 4 (22.2) |
| Incomplete adherence to treatment of previous TB infection | 5 (23.8) |
| Concomitant cyclophosphamide pulse, n (%) | 3 (14.3) |
| Concomitant steroid pulse, n (%) | 7 (33.3) |
| Mean steroid dose used during the prior 6 months, mg/day, mean (SD) ^†^ | 16.1 (16.9) |
| Time to develop TB, day, mean (SD) | 131.9 (92.2) |
| Initial steroid dose at baseline, mg/day, mean (SD) ^†^ | 65.0 (24.3) |
| Lymphopenia at baseline, n (%) | 6 (28.6) |
| Concomitant isoniazid treatment, n (%) | 2 (9.5) |

There were no tuberculosis cases in other rheumatic diseases during the observation.

^*^, including Takayasu’s arteritis (1) and polymyalgia rheumatica (1)

^†^, Based on the dose of prednisone.
