# Supplementary material for: Risk-benefit analysis of isoniazid monotherapy to prevent tuberculosis in patients with rheumatic diseases exposed to prolonged, high-dose glucocorticoids
Source: PLoS One. 2020 Dec 31;15(12):e0244239. doi: 10.1371/journal.pone.0244239 (PMC7774985; doi:10.1371/journal.pone.0244239)
Supplement: S3 Table — (DOCX) [file pone.0244239.s007.docx]

**S3 Table.** Clinical features of the 21 TB disease cases at diagnosis

| Case | Gender | Age | Underlying disease | Interval between baseline and TB (month) | Presence of high-risk factor at baseline | Steroid dose at baseline^†^ | Concomitant immunosuppressants at baseline | Steroid dose at TB^†^ | INH group | MDR-TB | Extrapulmonary TB | Anti-TB medication^*^ |
| --- | --- | --- | --- | --- | --- | --- | --- | --- | --- | --- | --- | --- |
| 1 | F | 31.8 | SLE | 10.1 | No | 60.0 | CYP pulse | 20.0 | No | No | No | HREZ |
| 2 | F | 32.7 | SLE | 2.2 | Yes | 50.0 | MMF | 60.0 | No | No | Yes | HREZ |
| 3 | F | 35.3 | EPGA | 4.0 | Yes | 35.0 | None | 30.0 | No | No | No | HREZ |
| 4 | F | 26.9 | SLE | 2.6 | No | 60.0 | Steroid pulse | 30.0 | Yes | Yes | No | RE+levofloxacin+streptomycin+PAS |
| 5 | F | 48.0 | SLE | 3.2 | No | 90.0 | None | 25.0 | No | No | No | HREZ |
| 6 | F | 51.0 | SLE | 1.2 | Yes | 30.0 | None | 40.0 | No | No | No | HREZ |
| 7 | F | 68.1 | SLE | 2.6 | No | 40.0 | Steroid pulse | 30.0 | No | No | No | HREZ |
| 8 | F | 41.8 | SLE | 2.9 | No | 30.0 | None | 15.0 | No | No | No | HREZ |
| 9 | F | 43.4 | SLE | 2.9 | Yes | 40.0 | None | 20.0 | No | Yes | No | Levofloxacin+amoxicilllin+PAS+cycloserine+prothionamide |
| 10 | F | 27.2 | Takayasu’s arteritis | 8.7 | Yes | 30.0 | None | 12.5 | No | No | Yes | HREZ |
| 11 | F | 35.9 | SLE | 2.9 | Yes | 40.0 | MMF | 20.0 | No | No | No | HREZ |
| 12 | F | 58.4 | PMR | 5.6 | No | 50.0 | None | 5.0 | Yes | No | No | HREZ |
| 13 | F | 21.2 | SLE | 11.4 | No | 80.0 | Steroid pulse | 20.0 | No | No | No | RE+levofloxacin+PAS |
| 14 | M | 31.6 | SLE | 1.8 | No | 60.0 | CYP pulse | 60.0 | No | Yes | No | Streptomycin+cycloserine+prothionamide+PAS |
| 15 | M | 34.6 | PAN | 7.8 | No | 60.0 | MTX | 50.0 | No | Yes | Yes | E+levofloxacin+streptomycin+PAS |
| 16 | F | 40.8 | Behcet’s disease | 2.1 | No | 30.0 | Cyclosporine | 10.0 | No | No | No | HRZ+levofloxacin |
| 17 | M | 61.0 | Dermatomyositis | 3.1 | No | 30.0 | None | 15.0 | No | No | No | HRZ+levofloxacin |
| 18 | F | 68.5 | SLE | 8.2 | Yes | 50.0 | None | 30.0 | No | No | Yes | HREZ |
| 19 | M | 24.3 | SLE | 3.8 | No | 60.0 | CYP pulse | 40.0 | No | No | Yes | HREZ |
| 20 | F | 30.9 | SLE | 0.7 | No | 90.0 | MMF | 30.0 | No | No | Yes | HREZ |
| 21 | M | 22.0 | SLE | 5.3 | No | 60.0 | Steroid pulse | 35.0 | No | No | Yes | HREZ |

CYP; cyclophosphamide; EGPA, eosinophilic granulomatosis with polyangiitis; INH, isoniazid; MDR, multidrug resistant; MMF, mycophenolic mofetil; MTX; methotrexate; PAN, polyarteritis nodosa; PMR, polymyalgia rheumatica; SLE, systemic lupus erythematosus; TB, tuberculosis;

^*^, H, isoniazid; R, rifampicin; E, ethambutol; P, pyrazinamide; PAS, para-aminosalicylate

^†^, based on the dose of prednisone, mg/day
