# Supplementary material for: Risk-benefit analysis of isoniazid monotherapy to prevent tuberculosis in patients with rheumatic diseases exposed to prolonged, high-dose glucocorticoids
Source: PLoS One. 2020 Dec 31;15(12):e0244239. doi: 10.1371/journal.pone.0244239 (PMC7774985; doi:10.1371/journal.pone.0244239)
Supplement: S4 Table — (DOCX) [file pone.0244239.s008.docx]

**S4 Table.** Clinical factors associated with AST/ALT elevation and peripheral neuropathy during INH treatment

|  | AST/ALT elevation | | Peripheral neuropathy | |
| --- | --- | --- | --- | --- |
|  | Univariable analysis  HR (95% CI) | Multivariable analysis ^a^  HR (95% CI) | Univariable analysis  HR (95% CI) | Multivariable analysis ^a^  HR (95% CI) |
| Age (per 5-year increment) | 1.14 (1.01 to 1.30) | 1.16 (1.02 to 1.32) | 1.01 (0.90 to 1.14) | ^b^ |
| Male sex | 1.03 (0.43 to 2.42) | ^b^ | 0.73 (0.26 to 2.05) | ^b^ |
| Systemic lupus erythematosus | 0.51 (0.22 to 1.18) | ^b^ | 0.59 (0.23 to 1.52) | ^b^ |
| High-risk subgroup | 1.41 (0.42 to 4.71) | ^b^ | NA ^c^ | ^b^ |
| Initial steroid dose at baseline (≥60 mg/day of prednisone vs. a lower dose) | 0.94 (0.43 to 2.05) | ^b^ | 0.42 (0.17 to 1.04) | 0.46 (0.18 to 1.18) |
| Concomitant oral cyclophosphamide | 0.74 (0.20 to 2.74) | ^b^ | 1.13 (0.30 to 4.29) | ^b^ |
| Concomitant cyclophosphamide pulse | 1.34 (0.53 to 3.41) | ^b^ | 0.50 (0.14 to 1.86) | ^b^ |
| Concomitant mycophenolate mofetil | 2.83 (0.45 to 17.83) | ^b^ | NA ^c^ | ^b^ |
| Concomitant cyclosporine | 2.24 (0.67 to 7.44) | ^b^ | 5.04 (1.44 to 17.60) | 4.63 (1.27 to 16.86) |
| Concomitant methotrexate | 2.60 (0.58 to 11.61) | ^b^ | 2.06 (0.39 to 11.06) | ^b^ |
| Concomitant steroid pulse | 1.26 (0.54 to 2.93) | ^b^ | 0.54 (0.20 to 1.51) | ^b^ |
| Mean steroid dose during the prior 6 months, mg/day | 1.05 (1.01 to 1.09) | 1.05 (1.01 to 1.10) | 1.02 (0.99 to 1.06) | ^b^ |
| Baseline lymphopenia | 1.14 (0.50 to 2.61) | ^b^ | 1.18 (0.45 to 3.08) | ^b^ |

ALT, alanine aminotransferase; AST, aspartate aminotransferase; CI, confidence interval; HR, hazard ratio;

^a^, Multivariable model included covariates with relevant association (P < 0.1) in the univariable analysis.

^b^, not included in the multivariable model

c, HR could not be calculated due to complete separation of the case.
