# Supplementary material for: Risk-benefit analysis of isoniazid monotherapy to prevent tuberculosis in patients with rheumatic diseases exposed to prolonged, high-dose glucocorticoids
Source: PLoS One. 2020 Dec 31;15(12):e0244239. doi: 10.1371/journal.pone.0244239 (PMC7774985; doi:10.1371/journal.pone.0244239)
Supplement: S5 Table — (DOCX) [file pone.0244239.s009.docx]

**S5 Table.** Incidence of TB disease and effect of INH treatment in the low-risk- and unknown-risk-subgroups

|  | Low-risk subgroup  (n = 124) | Unknown-risk subgroup (n = 1402) |
| --- | --- | --- |
| Control group, n | 114 | 1276 |
| INH group, n | 10 | 126 |
| Number of TB cases / Observation period (person-year) in the control group | 0 / 110.8 | 12 / 1251.4 |
| Number of TB cases / Observation period (person-year) in the INH group | 0 / 10.0 | 2 / 122.7 |
| Crude HR (95% CI) (vs. control group) | NA | 1.70 (0.38 to 7.56) |
| Adjusted HR (95% CI) (vs. control group)^a^ | NA | 0.89 (0.16 to 4.93) |

CI, confidence interval; HR, hazard ratio; INH, isoniazid; NA, non-applicable; TB, tuberculosis.

^a^ adjusted by SLE, duration of high-dose steroid treatment, concomitant steroid pulse, and previously used steroid dose.
