# Supplementary material for: Risk-benefit analysis of isoniazid monotherapy to prevent tuberculosis in patients with rheumatic diseases exposed to prolonged, high-dose glucocorticoids
Source: PLoS One. 2020 Dec 31;15(12):e0244239. doi: 10.1371/journal.pone.0244239 (PMC7774985; doi:10.1371/journal.pone.0244239)
Supplement: S1 Text — (DOCX) [file pone.0244239.s010.docx]

**S1 Text.** ICD-10 codes for detection of treatment episodes in patients with rheumatic diseases

Systemic lupus erythematosus (SLE, M32),

Systemic sclerosis (M34)

Dermatomyositis (M33.0, M33.1 and M33.9)

Polymyositis (M33.2)

Granulomatosis with polyangiitis (GPA, M31.3)

Microscopic polyangiitis (MPA, M31.7)

Eosinophilic granulomatosis with polyangiitis (EGPA, M30.1)

Polyarteritis nodosa (M30.0 and M30.8)

Rheumatoid arthritis (M05)

Adult-onset Still’s disease (AOSD, M06.1)

Behcet’s disease (M35.2)

Cryoglobulinemic vasculitis (D89.1)

Ankylosing spondylitis (M45)

Temporal arteritis (M31.5 and M31.6)

Polymyalgia rheumatica (M35.3)

Takayasu’s arteritis (M31.4)

Relapsing polychondritis (M94.1)

Sjogren syndrome (M35.0).
